# Supplementary figures and images for: High prevalence of atypical virulotype and genetically diverse background among Pseudomonas aeruginosa isolates from a referral hospital in the Brazilian Amazon
Source: PLoS One. 2020 Sep 10;15(9):e0238741. doi: 10.1371/journal.pone.0238741 (PMC7482967; doi:10.1371/journal.pone.0238741)

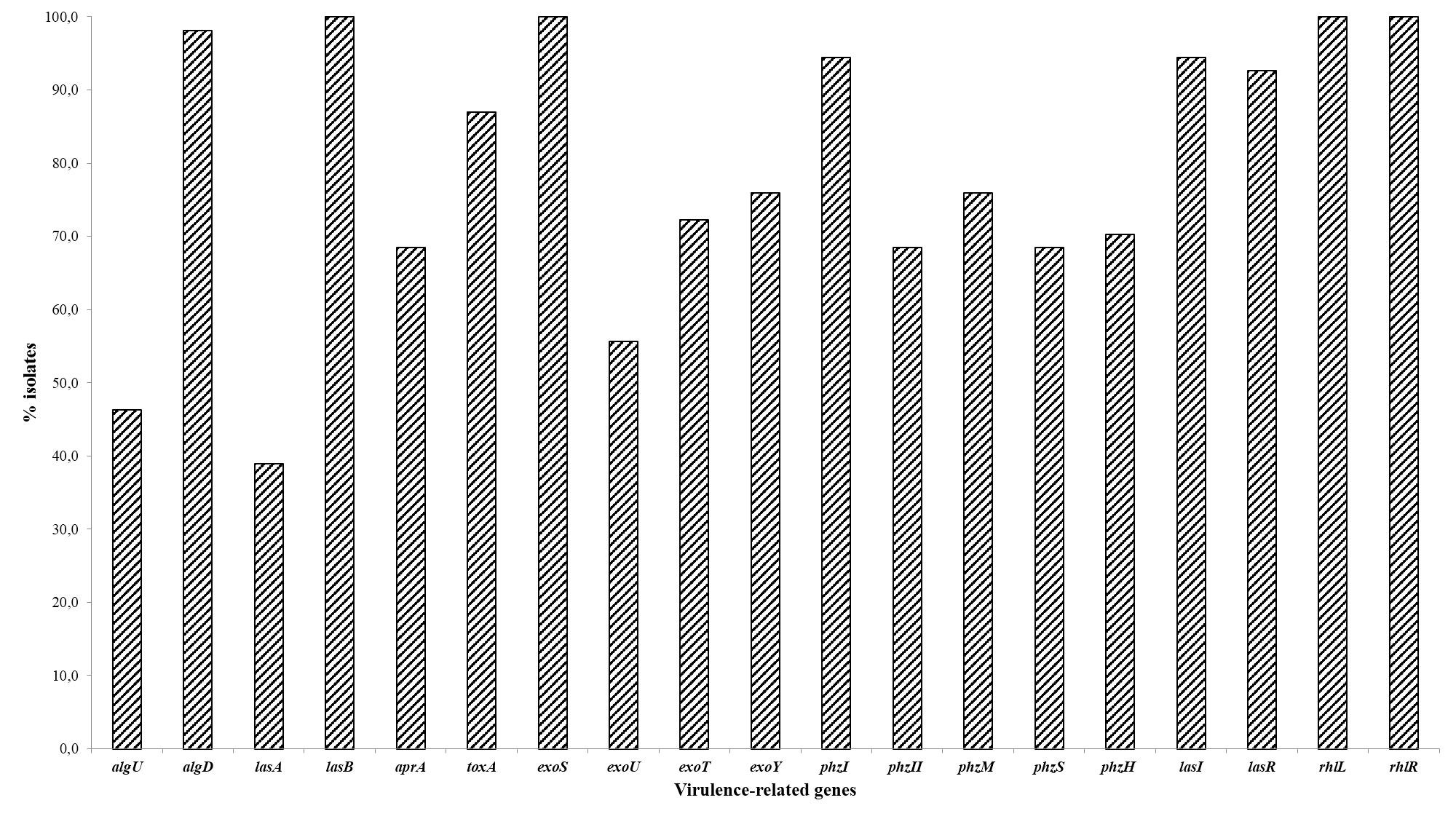

Supplement: S1 Fig — (TIF) [file pone.0238741.s002.tif]
