# Supplementary material for: High prevalence of atypical virulotype and genetically diverse background among Pseudomonas aeruginosa isolates from a referral hospital in the Brazilian Amazon
Source: PLoS One. 2020 Sep 10;15(9):e0238741. doi: 10.1371/journal.pone.0238741 (PMC7482967; doi:10.1371/journal.pone.0238741)
Supplement: Attachment — Submitted filename: PONE-D-20-15488_reviewer.1.pdf [file pone.0238741.s003.pdf]

PLOS ONE

High prevalence of atypical virulotype and genetically diverse background among Pseudomonas aeruginosa isolates from a referral hospital in the Brazilian Amazon

--Manuscript Draft--

**Manuscript Number:**	PONE-D-20-15488
**Article Type:**	Research Article
**Full Title:**	High prevalence of atypical virulotype and genetically diverse background among Pseudomonas aeruginosa isolates from a referral hospital in the Brazilian Amazon
**Short Title:**	Virulence, antimicrobial resistance and genotyping of <i>Pseudomonas aeruginosa</i>
**Corresponding Author:**	YAN CORREA RODRIGUES<br>Universidade do Estado do Para<br>Belém, Pará BRAZIL
**Keywords:**	Pseudomonas aeruginosa; virulence; exoU; antimicrobial resistance, genotyping; MLST
**Abstract:**	Pseudomonas aeruginosa is an opportunistic pathogen causing different types of infections, particularly in intensive care unit patients. Characteristics that favor its persistence artificial environments are related to its high adaptability, wide arsenal of virulence factors and resistance to several antimicrobials classes. Among the several virulence determinants, T3SS stands as the most important due to the clinical impact of exoS and exoU genes in patient's outcome. The molecular characterization of P. aeruginosa isolates helps in the comprehension of transmission dynamics and enhance knowledge of virulence and resistance roles in infection process. In the present study, we investigated virulence and resistance properties and the genetic background of P. aeruginosa isolated from ICUs patients at a referral hospital in Brazilian Amazon. A total of 54 P. aeruginosa isolates were characterized by detecting 19 virulence-related genes, antimicrobial susceptibility testing, molecular detection of β-lactamase-encoding genes and genotyping by MLST and rep-PCR. Our findings showed high prevalence of virulence-related markers, where 53.7% of the isolates presented at least 17 genes among the 19 investigated (P =0.01). The rare exoS + /exoU + cytotoxic virulotype was detected in 55.6% of isolates. Antimicrobial susceptibility testing revealed percentages of antibiotic resistance above 50% to carbapenems, cephalosporins and fluoroquinolones associated to MDR/XDR isolates. Isolates harboring both bla SPM-1 and bla OXA genes were also detected. Genotyping methods demonstrated a wide genetic diversity of strains spread among the different intensive care units, circulation of international MDR/XDR high-risk clones (ST111, ST235, ST244 and ST277) and emergence of seven novel MLST lineages. Finally, our findings highlight the spread of strains with high virulence potential and resistance to antimicrobials and may be useful on comprehension of pathogenicity process, treatment guidance and establishment of strategies to control the spread of epidemic P. aeruginosa strains.
**Order of Authors:**	Yan Corrêa Rodrigues
	Ismari Perini Furlaneto
	Arthur Henrique Pinto Marciel
	Ana Judith Pires Garcia Quaresma
	Eliseth Costa Oliveira de Matos
	Marília Lima Conceição
	Marcelo Cleyton da Silva Vieira
	Giulia Leão da Cunha Brabo
	Edilene do Socorro Nascimento Falcão Sarges
	Luana Nepomuceno Godim Costa Lima
	Karla Valéria Batista Lima

Powered by Editorial Manager® and ProduXion Manager® from Aries Systems Corporation

Additional Information:	
**Question**	**Response**
**Financial Disclosure**<br><br>Enter a financial disclosure statement that describes the sources of funding for the work included in this submission. Review the submission guidelines for detailed requirements. View published research articles from *PLOS ONE* for specific examples.<br><br>This statement is required for submission and **will appear in the published article** if the submission is accepted. Please make sure it is accurate.<br><br>**Unfunded studies**<br>Enter: *The author(s) received no specific funding for this work.*<br><br>**Funded studies**<br>Enter a statement with the following details:<br>• Initials of the authors who received each award<br>• Grant numbers awarded to each author<br>• The full name of each funder<br>• URL of each funder website<br>• Did the sponsors or funders play any role in the study design, data collection and analysis, decision to publish, or preparation of the manuscript?<br>• **NO** - Include this sentence at the end of your statement: *The funders had no role in study design, data collection and analysis, decision to publish, or preparation of the manuscript.*<br>• **YES** - Specify the role(s) played.<br><br>* typeset	This research was supported by funding from Fundação de Amparo à Pesquisa do Pará/Universidade do Estado do Pará (FAPESPA/UEPA) [Cooperation grant Nº004/2019], Programa Institucional de Bolsas de Iniciação Científica/Instituto Evandro Chagas - Conselho Nacional de Desenvolvimento Científico e Tecnológico (PIBIC/IEC-CNPq) and Instituto Evandro Chagas/Ministério da Saúde/Secretaria de Vigilância em Saúde (IEC/MS/SVS). The funders had no role in study design, data collection and analysis, decision to publish, or preparation of the manuscript.
**Competing Interests**<br><br>Use the instructions below to enter a competing interest statement for this submission. On behalf of all authors, disclose any competing interests that could be perceived to bias this work—acknowledging all financial support and any other relevant financial or non-financial competing interests.	The authors declare no potential conflict of interests regarding the publication of this study.

This statement **will appear in the published article** if the submission is accepted. Please make sure it is accurate. View published research articles from *PLOS ONE* for specific examples.<br><br>**NO authors have competing interests**<br><br>Enter: *The authors have declared that no competing interests exist.*<br><br>**Authors with competing interests**<br><br>Enter competing interest details beginning with this statement:<br><br>*I have read the journal's policy and the authors of this manuscript have the following competing interests: [insert competing interests here]*<br><br>* typeset	
**Ethics Statement**<br><br>Enter an ethics statement for this submission. This statement is required if the study involved:<br><br>• Human participants<br>• Human specimens or tissue<br>• Vertebrate animals or cephalopods<br>• Vertebrate embryos or tissues<br>• Field research<br><br>Write "N/A" if the submission does not require an ethics statement.<br><br>General guidance is provided below. Consult the submission guidelines for detailed instructions. **Make sure that all information entered here is included in the Methods section of the manuscript.**	No samples were collected for this study. Only stored samples were included without any contact and possibility of identifying the respective patients. Prior sampling and the present study were conducted in accordance with Helsinki Declaration and the Brazilian National Health Council and with approval of the ethics committee at Fundação Santa Casa de Misericórdia do Pará (referral hospital under study) (Nº CAAE 0086.0.440.000-10).

**Format for specific study types**<br><br>**Human Subject Research (involving human participants and/or tissue)**<br>• Give the name of the institutional review board or ethics committee that approved the study<br>• Include the approval number and/or a statement indicating approval of this research<br>• Indicate the form of consent obtained (written/oral) or the reason that consent was not obtained (e.g. the data were analyzed anonymously)<br><br>**Animal Research (involving vertebrate animals, embryos or tissues)**<br>• Provide the name of the Institutional Animal Care and Use Committee (IACUC) or other relevant ethics board that reviewed the study protocol, and indicate whether they approved this research or granted a formal waiver of ethical approval<br>• Include an approval number if one was obtained<br>• If the study involved *non-human primates*, add *additional details* about animal welfare and steps taken to ameliorate suffering<br>• If anesthesia, euthanasia, or any kind of animal sacrifice is part of the study, include briefly which substances and/or methods were applied<br><br>**Field Research**<br><br>Include the following details if this study involves the collection of plant, animal, or other materials from a natural setting:<br>• Field permit number<br>• Name of the institution or relevant body that granted permission	
**Data Availability**<br><br>Authors are required to make all data underlying the findings described fully available, without restriction, and from the time of publication. PLOS allows rare exceptions to address legal and ethical concerns. See the PLOS Data Policy and FAQ for detailed information.	Yes - all data are fully available without restriction

A Data Availability Statement describing where the data can be found is required at submission. Your answers to this question constitute the Data Availability Statement and **will be published in the article**, if accepted.<br><br>**Important:** Stating 'data available on request from the author' is not sufficient. If your data are only available upon request, select 'No' for the first question and explain your exceptional situation in the text box.<br><br>Do the authors confirm that all data underlying the findings described in their manuscript are fully available without restriction?	
**Describe where the data may be found in full sentences. If you are copying our sample text, replace any instances of XXX with the appropriate details.**<br><br>• If the data are **held or will be held in a public repository**, include URLs, accession numbers or DOIs. If this information will only be available after acceptance, indicate this by ticking the box below. For example: *All XXX files are available from the XXX database (accession number(s) XXX, XXX.).*<br>• If the data are all contained **within the manuscript and/or Supporting Information files**, enter the following: *All relevant data are within the manuscript and its Supporting Information files.*<br>• If neither of these applies but you are able to provide **details of access elsewhere**, with or without limitations, please do so. For example:<br><br>*Data cannot be shared publicly because of [XXX]. Data are available from the XXX Institutional Data Access / Ethics Committee (contact via XXX) for researchers who meet the criteria for access to confidential data.*<br><br>*The data underlying the results presented in the study are available from (include the name of the third party*	All relevant data are within the manuscript and its Supporting Information files.

and contact information or URL).<br>• This text is appropriate if the data are owned by a third party and authors do not have permission to share the data.<br><br>* typeset	
Additional data availability information:	

Powered by Editorial Manager® and ProduXion Manager® from Aries Systems Corporation

Manuscript
Click here to download Manuscript 2.Manuscript_22May2020.docx

High prevalence of atypical virulotype and genetically diverse background among *Pseudomonas aeruginosa* isolates from a referral hospital in the Brazilian Amazon

Yan Corrêa Rodrigues[1*], Ismari Perini Furlaneto[2], Arthur Henrique Pinto Marciel[3], Ana Judith Pires Garcia Quaresma[3], Eliseth Costa Oliveira de Matos[4], Marília Lima Conceição[1], Marcelo Cleyton da Silva Vieira[3], Giulia Leão da Cunha Brabo[3], Edilene do Socorro Nascimento Falcão Sarges[1], Luana Nepomuceno Godim Costa Lima[1,3], Karla Valeria Batista Lima[1,3*].

[1]Programa de Pós-graduação em Biologia Parasitária na Amazônia, Centro de Ciências Biológicas e da Saúde, Universidade do Estado do Pará (UEPA), Belém, Pará, Brazil.

[2]Programa de Pós-graduação em Educação em Saúde, Centro Universitário do Pará (CESUPA), Belém, Pará Brazil.

[3]Laboratorio de Biologia Molecular, Seção de Bacteriologia e Micologia, Instituto Evandro Chagas (IEC), Ministério da Saúde, Ananindeua, Pará, Brazil.

[4]Departamento de Patologia, Centro de Ciências Biológicas e da Saúde, Universidade do Estado do Pará (UEPA), Belém, Pará, Brazil.

*Corresponding authors:

E-mail: yan.13@hotmail.com (YCR)

E-mail: karlalima@iec.gov.br (KVBL)

Abstract

Pseudomonas aeruginosa is an opportunistic pathogen causing different types of infections, particularly in intensive care unit patients. Characteristics that favor its persistence artificial environments are related to its high adaptability, wide arsenal of virulence factors and resistance to several antimicrobials classes. Among the several virulence determinants, T3SS stands as the most important due to the clinical impact of *exoS* and *exoU* genes in patient's outcome. The molecular characterization of *P. aeruginosa* isolates helps in the comprehension of transmission dynamics and enhance knowledge of virulence and resistance roles in infection process. In the present study, we investigated virulence and resistance properties and the genetic background of *P. aeruginosa* isolated from ICUs patients at a referral hospital in Brazilian Amazon. A total of 54 *P. aeruginosa* isolates were characterized by detecting 19 virulence-related genes, antimicrobial susceptibility testing, molecular detection of β-lactamase-encoding genes and genotyping by MLST and rep-PCR. Our findings showed high prevalence of virulence-related markers, where 53.7% of the isolates presented at least 17 genes among the 19 investigated (P =0.01). The rare $exoS^{+}/exoU^{+}$ cytotoxic virulotype was detected in 55.6% of isolates. Antimicrobial susceptibility testing revealed percentages of antibiotic resistance above 50% to carbapenems, cephalosporins and fluoroquinolones associated to MDR/XDR isolates. Isolates harboring both $bla_{\mathrm{SPM\text{-}1}}$ and bla_{OXA} genes were also detected. Genotyping methods demonstrated a wide genetic diversity of strains spread among the different intensive care units, circulation of international MDR/XDR high-risk clones (ST111, ST235, ST244 and ST277) and emergence of seven novel MLST lineages. Finally, our findings highlight the spread of strains with high virulence potential and resistance to antimicrobials and may be useful on comprehension of pathogenicity

process, treatment guidance and establishment of strategies to control the spread of epidemic *P. aeruginosa* strains.

Introduction

Pseudomonas aeruginosa is an opportunistic pathogen, causing a wide spectrum of infections, including acute and chronic respiratory tract infections (RTIs) and bloodstream infections (BSIs) as the main infection sites [1–4]. It is estimated that *P. aeruginosa* is responsible to about 10% of all hospital-acquired-infections worldwide and associated to outbreaks in adult, pediatric and neonatal intensive care units (ICUs) due to the spread of multi-drug resistant (MDR) or extensively drug-resistant (XDR) and highly virulent *P. aeruginosa* strains, negatively impacting morbidity, mortality, length of stay and treatment costs of patients [5–7]. *P. aeruginosa* high adaptability, resistance to several antimicrobials classes and secretion of various virulence factors allows its persistence in artificial settings of high selective pressure, such as hospitals [8–10].

Pathogenicity in *P. aeruginosa* is multifactorial, relying on the regulation of virulence-related genes and expression of their respective factors, including adhesins, exotoxins, proteases and pigments. Virulence products may be passively secreted from bacterial cell or actively secreted via secretion systems, such as type I secretion system (T1SS), type II secretion system (T2SS) and type III secretion system (T3SS) [11–13]. T3SS is the most important and well characterized virulence determinant in *P. aeruginosa*, which allows the translocation of effector cytotoxic proteins into the host cell via a nano-syringe. So far, only four effector exotoxins encoded by the *exoS, exoU, exoT* and *exoY* genes have been identified, which are variably present and expressed by *P. aeruginosa* strains. ExoS and ExoU are associated to invasive and cytotoxic phenotypes, respectively, and rarely

concomitant detected, while ExoT and ExoY demonstrate few cytotoxic effects and are encoded by most of strains [8, 14, 15, 16, 17]. Furthermore, survey of cytotoxic/*exoU*[+] virulotype has been highly recommended due to the impact of this exotoxin in patient's mortality, especially in isolates from high risk settings, such as ICUs [19, 18].

The increasing trend of resistance to anti-pseudomonal antibiotics, especially carbapenems, aminoglycosides and fluoroquinolones has contributed to the emergence of *P. aeruginosa* MDR/XDR strains, posing a challenge to infection treatment [20–22]. Among the several resistance mechanisms in *P. aeruginosa*, the role of transferable resistance determinants is overwhelming, particularly those encoding classes A and D extended-spectrum β-lactamases (ESBLs) and class B metallo-β-lactamases (MBLs) [23–26].

Molecular typing of in-hospital isolates of *P. aeruginosa* contribute to the understanding of transmission and infection dynamics by strains exhibiting high degree of virulence and resistance, allowing the determination of genetic relationships between isolates, outbreaks identification, population structure studies and therapeutic management [27–29]. In spite of the polyclonal population structure of *P.* aeruginosa, several studies have shown a globally spread of strains denominated as high-risk clones (HRC), which are associated with most MDR/XDR isolates and specific virulence markers. Thus, characterization of *P. aeruginosa* isolates must be performed combining clonality evaluation with methods that can identify specific virulence and resistance determinants [19, 23, 31, 32].

The molecular characterization of local *P. aeruginosa* isolates is one of the first steps to understand the epidemiology of the pathogen at local and global level, helping to establish

surveillance strategies, reduce risk of outbreaks and enhance knowledge of virulence and resistance roles in infection process. Nonetheless, comprehensive epidemiological investigations on molecular level are still scarce, especially ones reporting data from Brazilian territory. In the present study we report in-depth data into the virulence, resistance properties and genetic diversity of in-patient ICU isolates from a referral hospital in the Brazilian Amazon region.

Material and Methods

Bacterial isolates

In this retrospective cross-sectional study, a total of 54 non-repetitive *P. aeruginosa* isolates stored in the Bacteriology and Mycology Section of the Evandro Chagas Institute were evaluated. Isolates were previously recovered of various clinical sources of patients hospitalized in different ICUs at a referral hospital, from January 2010 to June 2013 (S1 Table). Identification of isolates was performed on automated VITEK-2 system (bioMérieux, Marcy l'Etoile, France) and complemented by phenotypical and biochemical assays such as observation of colony morphology, Gram stain, oxidase test, absence of carbohydrate fermentation on triple sugar iron agar, oxidative behavior on Hugh Leifson medium and the cytochrome oxidase reaction.

DNA extraction and molecular detection of virulence-related genes

Genomic DNA was obtained from a single colony of overnight cultures for each *P. aeruginosa* isolate using UltraClean^{TM} Microbial DNA Isolation kit (MoBio

Laboratories, Carlsbad, CA, USA), following manufacturer's recommendations and quantified using the Picodrop PICO100 spectrophotometer (Picodrop Limited, Hinxton, UK). Adjusted DNA concentrations between 25ng – 50ng/µl were used for all subsequent molecular assays.

The detection of 19 virulence-related markers was performed by PCR: adhesion (*algU* and *algD*), T1SS (*aprA*), T2SS (*lasA*, *lasB* and *toxA*), T3SS (*exoS*, *exoU*, *exoT* and *exoY*), oxidative stress (*phzI*, *phzII*, *phzM*, *phzS* and *phzH*) and *quorum sensing* (QS)/regulation (*lasI*, *lasR*, *rhlL* and *rhlR*), using previously described primers and reaction parameters with slightly modifications [33–38]. Each PCR mixture was prepared in a final volume of 25µl, consisted of 1x PCR buffer (Qiagen, Hilden, Germany), 1x Q-solution (Qiagen, Hilden, Germany), 2mM of $MgCl_2$ (Qiagen, Hilden, Germany), 200 µM of each dNTP (Invitrogen™, São Paulo, Brazil), 0,5µM of each primer, 1U of HotStart Taq DNA polymerase and DNA template. Amplifications were performed on Veriti thermocycler (Applied Biosystems, Foster City, CA, USA), under the following conditions for all genes: 95ºC for 15 minutes; followed by 35 cycles at 95ºC for 1 minute, 60ºC for 45 seconds, 72ºC for 1 minute and a final extension step at 72ºC for 7 minutes. PCR products were subjected to electrophoresis on 1.5% agarose gel and amplicons visualized under ultraviolet light. *P. aeruginosa* ATCC 27853 and PA14 strains were used as positive control.

Antimicrobial susceptibility assays and definition of resistance phenotypes

Antimicrobial susceptibility testing determined the Minimum Inhibitory Concentration (MICs) by the broth microdilution method for six antimicrobial classes: cephalosporins

(cefepime – FEP), carbapenems (imipenem – IMP and meropenem – MER), penicilins + beta-lactamase inhibitor (piperacillin+tazobactam – TZP), aminoglycosides (gentamicin – GN), fluoroquinolones (ciprofloxacin – CIP) and polypeptides (polymyxin B – POL). All assays were performed in accordance to the Clinical and Laboratory Standards Institute guidelines and breakpoints [39] and isolates were classified as sensitive (S), intermediate (I) and resistant (R). Reference strains *Escherichia coli* ATCC 25922 and *P. aeruginosa* ATCC 27853 were used as quality control. For statistical analysis, isolates exhibiting resistant and intermediate susceptibility phenotypes were grouped as 'non-susceptible'. Isolates were phenotypically classified as MultiS if susceptible to all tested antimicrobial classes; Moderately resistant (ModR) if resistant to ≥1 drug in <3 antimicrobial classes; MDR if resistant to ≥1 drug in ≥3 antimicrobial classes and XDR if non-susceptible to 1 agent in all but ≤2 tested categories, according to previously described criteria [31, 40].

Molecular detection of antimicrobial resistance markers

The detection of β-lactamase-encoding genes, including ESBLs (*bla*$_{\text{CTX-M1}}$, *bla*$_{\text{OXA-2}}$, *bla*$_{\text{OXA-10}}$) and carbapenemases (*bla*$_{\text{IMP-1}}$, *bla*$_{\text{IMP-2}}$, *bla*$_{\text{VIM-1}}$, *bla*$_{\text{VIM-2}}$, *bla*$_{\text{SPM-1}}$, *bla*$_{\text{NDM-1}}$ and *bla*$_{\text{KPC-1}}$) was performed by PCR on Veriti thermocycler (Applied Biosystem, Foster City, CA, USA) as previously described [41–47]. PCR products were subjected to electrophoresis on 1.5% agarose gel and amplicons visualized under ultraviolet light.

Molecular typing based on MLST

MLST genotyping was performed according to protocol described by Curran et al. (2004) [48]. The seven housekeeping genes (*acsA*, *aroE*, *guaA*, *mutL*, *nuoD*, *ppsA*, and *trpE*) were amplified by PCR in a Veriti thermocycler (Applied Biosystems, Foster City, CA,

USA). Reaction products were bidirectionally sequenced using Big Dye Terminator v3.1 chemistry on ABI Prism 3100 Genetic Analyzer (Applied Biosystems, Foster City, CA, USA). Obtained results were compared to available data at PubMLST database (http://pubmlst.org/paeruginosa) to determine allelic profiles and sequence types (STs). Novel alleles and STs were submitted to PubMLST database for validation. PHYLOViZ 2.0 platform was used for data management and analysis of clonal complexes (CCs), which were defined by related ST clusters exhibiting variation in a single locus (single locus variants - SLV) or in two loci (double locus variants - DLV) [49].

Molecular typing based on rep-PCR

The genetic relatedness of isolates was investigated by semi-automated rep-PCR on DiversiLab™ Strain Typing System (bioMérieux, Marcy-L'Étoile, France) using the DiversiLab *P. aeruginosa* kit (Bacterial Barcodes, bioMérieux, Marcy-L'Étoile, France), according to the manufacturer's instructions. Fingerprints were obtained by electrophoresis using microfluidic lab-on-a-chip on Agilent 2100 Bioanalyzer equipment (Agilent Technologies, Palo Alto, CA, USA) and analysis performed with DiversiLab on-line software (v 3.4) applying the Pearson correlation coefficient. Isolates were classified as in the same clonal group (genotypically indistinguishable) if the similarity was ≥97%, and as unique pattern if the similarity was <97%.

Statistical analysis

The G-test of independence or Fisher's exact test was applied to verify the association between resistance, virulence markers, isolation sites, clonal groups and STs; standardized residuals and adjusted residuals was used as a post-hoc test after a statistically significant G-test of independence. The distribution of virulence-related

genes among isolates was verified by the Lilliefors test. Values of $P \leq 0.05$ were considered statistically significant. All analyzes were performed using the statistical software BioEstat® 5.4 [50].

Ethics statement

No samples were collected for this study. Only stored samples were included without any contact and possibility of identifying the respective patients. Prior sampling and the present study were conducted in accordance with Helsinki Declaration and the Brazilian National Health Council [51] and with approval of the ethics committee at Fundação Santa Casa de Misericórdia do Pará (referral hospital under study) (Nº CAAE 0086.0.440.000-10).

Results

Bacterial isolates and distribution of virulence-related genes

This study evaluated 54 *P. aeruginosa* isolates distributed in the adult ICU (AICU, 28/54 –51.8%), followed by pediatric ICU (PICU, 14/54 – 26.0%) and neonatal ICU (NICU, 12/54 – 22.2%). Regarding clinical origin, isolates were obtained from RTI (23/54 – 42.6%), BSI (14/54 – 25.9%) and others various sources (17/54 –31.5%), including catheter (n= 6), rectal swab (n=3), surgical wound (n=3), ocular secretion (n=2), gastric secretion (n=1), urethral secretion (n=1), urine (n=1) (S1 Table).

Evaluated isolates harbored at least five virulence-related genes and seven isolates harbored all 19 investigated genes. In addition, a heterogeneous distribution of genes was observed, where 53.7% of the isolates presented at least 17 genes among the 19 investigated (P =0.01). The lowest detection frequencies were observed for *lasA* (21/54

– 38.9%) and *algU* (25/54 – 46.3%) genes, while *lasB*, *exoS*, *rhlL* and *rhlR* genes were detected in all isolates (54/54 – 100%) (S1 Fig and S1 Table). Definition of virulotypes was based on the detection of T3SS *exoS/exoU* genes, where it was observed the presence of invasive/cytotoxic ($exoS^+/exoU^+$; 30/54 – 55.6%) and invasive ($exoS^+/exoU^-$; 24/54 – 44.4%) virulotypes. $exoS^+/exoU^+$ virulotype and *phzH* gene were detected with significantly lower frequency at NICU (P =0.0461 and p= 0.0491, respectively), and among isolates from BSI (P =0.0027 and P =0.0244, respectively), as well as *exoY* gene (P =0.0435) (Tables 1 and 2).

Table 1. Distribution of virulence-related genes according to patient's ICUs.

Related function	Gene	AICU *n* = 28	PICU n = 14	NICU *n* = 12	*P*-value*
Adhesion	*algU*	12	7	6	0.8758
	algD	28	13	12	0.4601
T1SS	*aprA*	21	10	6	0.3204
T2SS	*lasA*	9	7	5	0.5379
	lasB	28	14	12	1.000
	toxA	23	13	11	0.9329
T3SS	*exoS*$^{+}$/ *exoU*$^{+}$	17	10	3[a]	0.0461*
	exoS^{+}/ *exoU*$^{-}$	11	4	9	
	exoT	21	12	6	0.1371
	exoY	23	12	6	0.0863
Oxidative stress	*phzI*	27	13	11	0.8346
	phzII	21	11	5	0.0959
	phzM	22	12	7	0.2775
	phzS	20	12	5	0.0581
	phzH	21	12	5[a]	0.0491*
QS/regulation	*lasI*	26	13	12	0.5386
	lasR	26	12	12	0.3334
	rhlL	28	14	12	1.000
	rhlR	28	14	12	1.000

T1SS, type I secretion system; T2SS, type II secretion system; T3SS, type III secretion system; QS, *quorum sensing*; AICU, adult intensive care unit; PICU, pediatric intensive care unit; NICU, neonatal intensive care unit.

* *P* values were calculated using the G-test of independence. [a] Frequency lower than expected at random.

Table 2. Distribution of virulence-related genes according to the *P. aeruginosa* isolates clinical sources.

Related function	Gene	RTI n = 23	BSI n = 14	Other n = 17	*P*-valor*
Adhesion	*algU*	12	6	7	0.7617
	algD	23	14	16	0.5003
T1SS	*aprA*	15	7	15	0.0618
T2SS	*lasA*	7	5	9	0.3569
	lasB	23	14	17	1.000
	toxA	21	13	15	0.9132
T3SS	$exoS^{+}$/ $exoU^{+}$	13	3[a]	14	0.0027*
	$exoS^{+}$/ $exoU^{-}$	10	11	3	
	exoT	17	7	15	0.0670
	exoY	19	7[a]	15	0.0435*
Oxidative stress	*phzI*	23	13	15	0.2276
	phzII	17	7	13	0.2489
	phzM	18	8	15	0.1449
	phzS	17	6	14	0.0596
	phzH	17	6[a]	15	0.0244*
QS/regulation	*lasI*	21	13	17	0.3815
	lasR	21	13	16	0.9518
	rhlL	23	14	17	1.000
	rhlR	23	14	17	1.000

T1SS, type I secretion system; T2SS, type II secretion system; T3SS, type III secretion system; RTI, respiratory tract infection; BSI, bloodstream infection; Other: catheter (n= 6), rectal swab (n=3), surgical wound (n=3), ocular secretion (n=2), gastric secretion (n=1), urethral secretion (n=1), urine (n=1).

* *P* values were calculated using the G-test of independence. [a] Frequency lower than expected at random.

Antimicrobial susceptibility features

Antimicrobial susceptibility testing revealed that isolates were mainly non-susceptible to carbapenems (IMP and MER; 36/54 – 66.7%), followed by FEP (27/54 – 50.0%), CIP (27/54 – 50.0%), GN (24/54 – 44.4%) and TZP (17/54 – 31.5%). All isolates were susceptible to POL (S1 Table). According to phenotypical classification, most of the isolates were classified as ModR (23/54 – 42.6%), followed by XDR (16/54 – 29.6%), MultiS (9/54 – 16.7%) and MDR (6/54 – 11.1%). MDR/XDR were significantly predominant in the AICU (Table 3) (*P* = 0.0003). There was no significant association between the presence of T3SS virulotypes and antimicrobial resistance (Table 4), where 33.3% (18/30) $exoU^{+}$ were classified as MultiS or ModR.

Table 3. Distribution of *P. aeruginosa* isolates according to susceptibility phenotype among the ICUs of in reference hospital.

	**MultiS/ModR**	**MDR/XDR**	***P*-value***
**ICU**			
AICU	11	17	
PICU	9	5	0.0003
NICU	12	0	

ICU, intensive care unit; AICU, adult intensive care unit; PICU, pediatric intensive care unit; NICU, neonatal intensive care unit; MultiS, susceptible to all tested antibiotics; ModR, moderately resistant; MDR, multi-drug resistant; XDR, extensively drug-resistant.
* *P* value was calculated using the G-test of independence.

Table 4. Distribution of T3SS virulotypes according to antimicrobial susceptibility of *P. aeruginosa* isolates.

	exoS^{+}/***exoU***$^{+}$ ***n* = 30**	***exoS***$^{+}$ /***exoU***$^{-}$ ***n* = 24**	***P*-value***
**Antimicrobial resistance**			
FEP	15	12	1.000
IMP	21	15	0.5770
MER	15	11	0.7905
TZP	9	8	1.000
GN	13	11	1.000
CIP	14	13	0.7846
**Susceptibility phenotype**			
MultiS	3	6	0.2702
ModR	15	8	0.2738
MDR	5	1	0.2100
XDR	7	9	0.3695
MDR+XDR	12	10	1.000

FEP, cefepime; IMP, imipenem; MER, meropenem; TZP, piperacillin+tazobactam; GN, gentamicin; CIP, ciprofloxacin; POL, polymyxin B; MultiS, susceptible to all tested antibiotics; ModR, moderately resistant; MDR, multi-drug resistant; XDR, extensively drug-resistant.
* *P* values were calculated using the Fisher's exact test.

Regarding molecular survey of antimicrobial resistance genes, 20.4% (11/54) of *P. aeruginosa* isolates harbored *bla*$_{CTX-M1,}$ followed by *bla*$_{SPM-1}$ (5/54 – 9.2%), *bla*$_{OXA-2}$ (3/54 – 5.5%) and *bla*$_{OXA-10}$ (3/54 – 5.5%). Two isolates harbored both *bla*$_{SPM-1}$ and *bla*$_{OXA-2}$ and *bla*$_{SPM-1}$ and *bla*$_{OXA-10}$ genes. One isolate harbored *bla*$_{OXA-2}$ gene and one isolate harbored *bla*$_{OXA-10.}$ Four isolates harboring *bla*$_{SPM-1}$ genes and seven isolates harboring *bla*$_{CTX-M1}$ were classified as XDR. The *bla*$_{IMP}$, *bla*$_{VIM}$, *bla*$_{NDM}$ and *bla*$_{KPC}$ genes were not detected.

Molecular typing based on MLST

MLST genotyping revealed a highly diverse genetic background with the presence of 22 different STs, including seven novel STs (ST2524, ST2541, ST2552, ST2554, ST2555, ST 2556 and ST2603) and 15 STs previously reported (ST111, ST170, ST235, ST244, ST277, ST360, ST463, ST500, ST508, ST1076, ST1197, ST1284, ST1655, ST2100 and ST2437). Novel allele 218/*aroE* was associated to ST2603. Genetic relationship analysis demonstrated the presence founders STs of four HRC CC, including CC/ST111 (2/22 – 9.0%), CC/ST235 (5/22 – 22.7%), CC/ST244 (9/22 – 40.9%) and CC/ST277 (6/22 – 27.2%). Among the seven novel STs, ST 2554 emerged as DLV of CC/ST274, which is a HRC; ST 2555 as SLV of ST 316 and the other five STs (ST2524, ST2541, ST2552, ST 2556 and ST2603) were singletons.

Among the isolates, 40.7% (22/54) were associated to HRC and 59.3% (32/54) to non-HRC. HRC were significantly predominant in the AICU (*P* =0.0100) (Table 5), especially ST244 (8/28 – 28.5%). Although no significant association between MLST genotypes and T3SS virulotypes was observed, 50% (11/22) of HRC presented $exoS^{+}/exoU^{+}$ virulotype (*P* =0.5820) (Table 5). Non-susceptibility to FEP, IMP, MER, GN, CIP (*P* < 0.001) and TZP (*P* =0.0198) was predominant among HRC strains (Table 5). All 16 XDR isolates were associated to the detected HRC (*P* <0.0001) (Table 5) and only one MDR isolate belonged to non-HCR (ST2524); whereas all ModR isolates were associated to non-HRC (*P* <0.0001) (Table 5) and only one MultiS isolate belonged to HCR (ST244) (Fig 1). Five isolates harboring bla_{SPM-1}, *bla*OXA_{-2} and bla_{OXA-10} genes belonged to HRC ST277 and one isolate harboring only *bla*OXA_{-2} was associated to non-HRC ST508;

while isolates harboring *bla*$_{CTX-M1}$ belonged to HRC ST111, ST244, ST253 (10/11) and to non-HRC ST1284 (1/11).

Fig 1. Minimum spanning tree of the 54 *P. aeruginosa* isolates from the referral hospital in the Brazilian Amazon genotyped by MLST.

Each circle represents a different ST and the size of the circle is proportional to the number of isolates related to the respective ST. Each of the 4 susceptibility phenotypes is represented by a different color. Dotted lines represent multilocus variants. The graph shows the percentages of HRC associated with a particular susceptibility phenotype.

Table 5. Distribution of HRC and non-HRC according to ICUs, T3SS virulotypes and antimicrobial susceptibility of *P. aeruginosa* isolates.

	**HRC** *n* = 22	**non-HRC** *n* = 32	***P*-value***
**ICU**			
AICU	16[a]	12[b]	
PICU	5	9	0.0100
NICU	1[b]	11[a]	
**T3SS Virulotype**			
exoS+/exoU+	11	19	0.5820
exoS+/exoU-	11	13	
**Antimicrobial resistance**			
FEP	18	9	0.0002
IMP	20	16	0.0027
MER	17	9	0.0007
TZP	11	6	0.0198
GN	20	4	< 0.0001
CIP	21	6	< 0.0001
**Susceptibility phenotype**			
MultiS	1	8	0.0676
ModR	0	23	< 0.0001
MultiS+ModR	1	31	< 0.0001
MDR	5	1	0.0706
XDR	16	0	< 0.0001
MDR+XDR	21	1	< 0.0001

HRC, high-risk clones; ICU, intensive care unit; AICU, adult intensive care unit; PICU, pediatric intensive care unit; NICU, neonatal intensive care unit; T3SS, type III secretion system; FEP, cefepime; IMP, imipenem; MER, meropenem; TZP, piperacillin+tazobactam; GN, gentamicin; CIP, ciprofloxacin; POL, polymyxin B; MultiS, susceptible to all tested antibiotics; ModR, moderately resistant; MDR, multi-drug resistant; XDR, extensively drug-resistant.

* *P* values were calculated using the G-test of independence or Fisher's exact test.

[a] Frequency higher than expected at random. [b] Frequency lower than expected at random.

Molecular typing based on rep-PCR

Genotyping by rep-PCR on DiversiLab™ System revealed 36 distinct fingerprint patterns, of which 26 were unique patterns and ten clonal groups comprised of two to eight isolates (Fig 2). Strains associated to clonal groups were found circulating in different ICUs, however, no statistically significant association was observed between clonality and ICUs (P =0.5063) (Table 6). The frequency of virulotypes was similar between the different clonal patterns defined by DiversiLab™ (P =1.000) (Table 6). MDR/XDR isolates were found in higher proportion among clonal groups strains (P =0.0141) (Table 6). Isolates harboring bla_{SPM-1}, *bla*OXA$_{-2}$ and bla_{OXA-10} genes were related to clonal groups A, B and C/ST277, while bla_{CTX-M1} gene was distributed among clonal groups (D, E, I and H) and unrelated strains (Fig 2). Genetic diversity observed through MLST genotyping presented a good association with fingerprint diversity found by rep-PCR, of which seven clonal groups were associated to HRC, but without reaching significant association (P =0.0573) (Table 6).

Fig 2. Dendrogram of genetic similarity of the 54 *P. aeruginosa* isolates from the referral hospital in the Brazilian Amazon genotyped by rep-PCR in the DiversiLab™ System.

* Novel STs. ST, sequence type; ICU, intensive care unit; AICU, adult intensive care unit; PICU, pediatric intensive care unit; NICU, neonatal intensive care unit; RTI, respiratory tract infection; BSI, bloodstream infection; T3SS, type III secretion system; MultiS, susceptible to all tested antibiotics; ModR, moderately resistant; MDR, multi-drug resistant; XDR, extensively drug-resistant.

Table 6. Distribution of rep-PCR patterns according ICUs, T3SS virulotypes and antimicrobial susceptibility of *P. aeruginosa* isolates.

	**Clonal Group** *n* **= 28**	**Unique patterns** *n* **= 26**	***P*-valor***
**ICU**			
AICU	14	14	
PICU	9	5	0.5063
NICU	5	7	
**T3SS Virulotype**			
exoS+/exoU+	16	14	1.000
exoS+/exoU-	12	12	
**Susceptibility phenotype**			
MultiS	3	6	0.2863
ModR	9	14	0.1683
MultiS+ModR	12	20	0.0141
MDR	5	1	0.1938
XDR	11	5	0.1410
MDR+XDR	16	6	0.0141
**MLST**			
HRC	15	13	0.0573
non-HRC	7	19	

AICU, adult intensive care unit; PICU, pediatric intensive care unit; NICU, neonatal intensive care unit; T3SS, type III secretion system; MultiS, susceptible to all tested antibiotics; ModR, moderately resistant; MDR, multi-drug resistant; XDR, extensively drug-resistant; HRC, high-risk clones.

* *P* values were calculated using the G-test of independence or Fisher's exact test.

Discussion

P. aeruginosa is currently one of the most relevant opportunistic pathogens causing acute infections, particularly among patients admitted to ICUs [5–6]. The occurrence of infection episodes and mortality in ICUs is significantly higher compared to other hospital wards; this scenario is justified by the complexity of the patients' clinical condition, such

as immunosuppression and pre-existing diseases, use of invasive diagnostic and therapeutic maneuvers, as well as, the spread of epidemic and highly virulent strains of *P. aeruginosa* [2, 4, 23, 32]. *P. aeruginosa* isolates included in our study were mainly recovered from RTI (42.6%) and BSI (25.9%) of patients admitted to different ICUs, similarly as previously reported infections frequencies, which varied between 9% to 35% in adult ICUs, reaching 62% in neonatal and pediatric ICUs and with RTI and BSI as major clinical sources [2, 3, 4, 6, 52, 53].

Although considered an opportunistic pathogen, the myriad of virulence products of *P. aeruginosa* may severally impact patients' clinical condition. The combination of multiple genes and virulence factors tends to affect pathogenesis and determine the outcome of an infectious process, and depending on the location and type of infection, the importance and role of a given factor may be different [10, 38, 54]. The detection of virulence-related markers revealed a high prevalence of genes, with detection of at least five genes per isolate and 17 of the 19 genes investigated in more than 50% of the isolates (P =0.01), demonstrating a high virulence potential of our strains (S1 Fig). Although our findings are consistent with several other reports that indicates a high prevalence and conserved nature of the *P. aeruginosa* genome regarding the presence of virulence-related genes, variations in virulence patterns are observed in isolates from different geographic areas and settings, highlighting the need to investigate different markers among distinct *P. aeruginosa* populations [1, 13, 23, 35, 55, 56].

The *algD* gene was detected in 98.1% of isolates, while absence of *algU* was noted in more than 53.0%. These findings differ from those reported by Hassuna et al (2020) [1] and Fazeli et al (2014) [57], who observed higher frequencies for *algU* and lower frequencies for *algD*, respectively. As both genes encode fundamental proteins for

alginate biosynthesis, functioning as one of its main adhesins and mainly found in the respiratory tract of patients with acute infections and cystic fibrosis (CF), the low frequency of the *algU* may be associated to the high frequency of non-RTI isolates (57.4%) and also indicate deficient alginate production by most of our isolates [10, 58].

The high prevalence of the *aprA* gene is in line with other studies [1, 13, 56, 59], highlighting the importance of zinc-metalloprotease secreted by T1SS, which has functions related to invasion, causing collagen and fibrinogen degradation in synergy with T2SS elastases, and evasion of the immune system through the inactivation of several cytokines, contributing to bacterial survival [60–61]. Three proteases - two elastases (LasA and LasB) and an exotoxin (ExoA) - secreted by *P. aeruginosa* are constituents of T2SS. The detection frequencies of 87.0% for *toxA* and 100.0% for *lasB,* in addition to the absence *lasA* among more than 60% our strains are in agreement with several other studies [1, 13, 56, 57, 62, 63]. Elastases act synergistically and have high elastolytic capacity mainly in blood vessels and lung tissues, while ExoA inhibits host protein synthesis leading to cell death [64–65]. Thus, our results reinforce the hypothesis of a significantly higher prevalence of *lasB* over *lasA* in *P. aeruginosa* clinical isolates [37, 66].

The phenazine compounds encoded by the *phzI, phzII, phzH, phzM* and *phzS* genes have siderophoric activity, cause an increase in oxidative stress and mitochondrial inactivation [10, 67]. Interestingly, Bradbury et al (2010) [13] correlated a high prevalence of these genes to *P. aeruginosa* clinical isolates, disagreeing with the low prevalence reported among our strains, and mainly of *phzH* gene, which absence was significant among isolates from BSI (*P* =0.0244) at NICU (*P* =0.0491) (Tables 1 and 2).

The QS genes were detected at frequencies above 92.0%, however, four isolates did not harbor *lasR* gene and three isolates *lasI* gene. The QS system in *P. aeruginosa* is coordinated by the *las* and *rhl* systems that function hierarchically for bacterial survival in the face of environmental changes, biofilm formation and control of several other virulence factors [10, 12]. Although a decrease in the ability to express virulence factors has been reported due to the absence and/or mutations in genes related to such QS, this does not necessarily compromise the ability to cause infection due to regulation mediated by the *rhl* system [12, 68, 69] Our results are in agreement with a study carried out by Senturk et al. (2012) [12] and Ruiz-Roldán et al. (2018) [56]; however, disagreeing with Aboushleib et al. (2015) [69], who reported gene deficiency in more than 60% in clinical isolates.

T3SS is the most important virulence marker in *P. aeruginosa*. The *exoS, exoU, exoT* and *exoY* genes encode toxins which are injected directly into the cytosol of the host cells and have functions related to anti-phagocytosis, necrotic and cytotoxic damage by a phospholipase, prevention of healing process and edema formation, respectively [10, 16, 54]. T3SS genes are variably present in isolates of *P. aeruginosa*, being the chromosomal genes *exoS, exoT* and *exoY* distributed among almost all strains [16, 18, 23]. T3SS *exoS, exoT* and *exoY* genes were detected in 100%, 72.2% and 75.9%, respectively. *exoS* results are in accordance with the hypothesis of a wider distribution of this gene due to its chromosomal nature. Nevertheless, the fact of in nearly 30% of our isolates *exoT* and *exoY* genes were not detected and a significant absence of *exoY* gene among BSI isolates (P =0.0435) (Table 2) demonstrate a contrary trend to the hypothesis of their universal distribution among *P. aeruginosa* strains, as well as, several studies that rarely report absence of these genes [13, 15, 16, 55, 56, 70].

The *exoU* gene is found less frequently due to its presence being associated to the PAPI-2 in the accessory genome of *P. aeruginosa*, however, its presence and the secretion of its respective protein are markers of very highly cytotoxic phenotype and associated with several types of acute infections, antimicrobial resistance phenotype, polymicrobial infections and early mortality individuals [18, 31, 71, 72]. Moreover, *exoS/exoU* genes tend to be mutually exclusive, being observed a higher prevalence of the invasive virulotype (*exoS*$^{+}$/*exoU*$^{-}$) among *P. aeruginosa* strains worldwide [16, 19, 23, 59]. Interestingly, 55.6% of our isolates presented the invasive/cytotoxic virulotype (*exoS*$^{+}$/*exoU*$^{+}$). Highlighting the rare occurrence of this phenomenon, there are only a few studies reporting a high proportion of this virulotype among *P. aeruginosa* hospital isolates [15, 35, 55]. We also observed a significant absence of *exoS*$^{+}$/*exoU*$^{+}$ virulotype among BSI isolates (p= 0.0027) and in the NICU (p= 0.0461), ward in which occurred the majority of BSI episodes among admitted patients (10/12 – 83.3%) (Tables 1 and 2), contradicting the important association established by several authors between the presence of *exoS*$^{+}$/*exoU*$^{+}$ virulotype among BSI isolates [18, 55, 73, 74]. Despite reports, the concomitant presence of genes still is not well understood, considering: (I) absence of a direct relationship between the genes, where *exoS*, *exoT* and *exoY* are found in the core genome of *P. aeruginosa*, while *exoU* is found associated to the PAPI-2 in the accessory genome; (II) the impact on bacterial fitness due to the presence of both genes, which would stimulate the loss of one of them; and (III) the production of both toxins could generate a higher immune response, making the pathogen less capable of establishing infection due to its high immunogenicity [13, 16, 19, 23, 71].

Our findings revealed that more of 66% of our isolates were resistant to carbapenems, followed by high resistance to cephalosporins, fluoroquinolones and aminoglycosides.

Moreover, 40.7% of our isolates were classified as MDR or XDR, which were significantly predominant in the AICU (*P* =0.0003) (Table 3) and associated to genetically related clonal groups (*P* =0.0141) (Table 6). For instance, our findings agree with recent data reported by Santos et al. (2019) [20], which also showed an increasing trend on resistance carbapenems, aminoglycosides and fluoroquinolones associated to spread of *P. aeruginosa* MDR/XDR strains over 21 years' period in Rio de Janeiro, Brazil. Others studies conducted in Brazil and Latin America also revealed high resistance rates to cephalosporins, fluoroquinolones and aminoglycosides [75–78]. These findings emphasize the growing global trend on antibiotic resistance been reported by several studies and rises concern, especially in Latin America countries, where carbapenems still remains as the main choice for the treatment of infections by MDR *P. aeruginosa* [21, 22, 23, 79, 80]. A reduced number of transmissible resistance markers were detected among our isolates, with *bla*$_{\text{CTX-M1}}$ (20.4% – 11/54) as the most frequent, followed by *bla*$_{\text{SPM-1}}$ (5/54 – 9.2%), *bla*$_{\text{OXA-2}}$ (3/54 – 5.5%) and *bla*$_{\text{OXA-10}}$ (3/54 – 5.5%). Our data demonstrate the predomination and wide spread of SPM-1 MDR/XDR strains in Brazilian hospitals and with the increasing transmission trend of ESBLs CTX-M and OXA variants genes worldwide, as previously reported [20, 23, 45, 76, 81, 82]. Finally, our results highlight the impact of horizontally acquired resistance mechanisms, mainly ESBLs and class B MBL, in the increasing resistance rates and its commonly association to MDR/XDR *P. aeruginosa* strains [18, 21, 23, 79].

The interaction between virulence and antibiotic susceptibility has been subject of several studies, suggesting that these relationships are antagonistic, since the presence of resistance mechanisms can determine a compromising biological cost for bacterial virulence and vice versa. In fact, the acquisition of resistance genes through the

incorporation of mobile genetic elements in the bacterial chromosome can lead to the inactivation of genes involved in the production of some virulence factors and decreased cytotoxicity [23, 72, 74, 83]. Nevertheless, other authors suggest that *exoU*$^{+}$ virulotype is associated resistance to fluoroquinolones and carbapenems, pointing out that the relationship between virulence and resistance may also occur in a synergistic sense, especially in high antibiotic pressure settings, such as ICUs [9, 13, 15, 16, 84]. In spite of that, our data do not suggest this association, as the presence of T3SS virulotypes was not related to any antimicrobial class or resistance phenotype (Table 4). Yet, 40% of *exoS*$^{+}$/*exoU*$^{+}$ isolates were associated to MDR/XDR phenotype, rising concern as a high prevalence of *exoS*$^{+}$/*exoU*$^{+}$ could facilitate the virulotype transmission to other MDR/XDR isolates, worsening the clinical scenario in the growing trend dissemination highly virulent MDR/XDR strains.

MLST revealed the presence of 22 different STs, including four different HRC (ST111, ST235, ST244 and ST277), seven newly identified STs and a novel allele related to *aroE* gene, demonstrating the high genetic diversity and an absence of relationship among most of our studied *P. aeruginosa* strains. Such findings are also in line with those obtained by rep-PCR genotyping, which revealed the presence of 36 fingerprints, 26 of which are unrelated (Figs 1 and 2). This high genetic diversity may be explained as the study site is a regional reference hospital for several specialties. It is likely that inter-hospital transit patients and/or who spend short periods in the hospital environment, as well as healthcare professionals, carry isolates and insert them in the hospital environment, and consequently causing infections in ICU patients because of numerous risk factors, such as immunosuppression conditions and virulence potential of isolates. Our data also corroborate the hypothesis of epidemic non-clonal population structure of *P. aeruginosa*,

indicating the dispersion of global HRC in hospitals settings and the continuous description of new *P. aeruginosa* STs worldwide [15, 23, 55, 56, 76, 85, 86].

Contrary to the predominance in other studies, HRC were associated with only 40% of our isolates [18, 23]. However, such strains demonstrate significantly association MDR/XDR phenotypes, carriage of resistance markers and persistence in ICUs (Table 5). ST111 has been detected in all continents [23, 31] and in our study was associated to clonal group I, composed by two MDR/XDR from different periods, of which only one harboring was *bla*$_{\text{CTX-M1}}$ gene, suggesting the latter acquisition of this marker (Fig 2). ST235 is the most widely spread *P. aeruginosa* HRC associated MDR/XDR isolates and carriage various resistance markers [23, 87]. Of the five ST235 isolates were MDR/XDR, of which three comprised the clonal group D, dispersed in different ICUs and carrying the gene *bla*$_{\text{CTX-M1}}$ gene, and two unrelated isolates, of which one also harboring *bla*$_{\text{CTX-M1}}$ gene (Fig 2). Kos et al. (2015) [88] has also detected the presence of CTX-M β-lactamase in Brazilian ST235 isolates. ST244 also has a worldwide distribution, being associated to carriage of different resistance markers, but not always related to MDR/XDR isolates [23, 88, 89, 90]. High frequencies were also reported by Brazilian studies evaluating clinical and environmental isolates [20, 76, 91]. ST244 was the most prevalent HRC among our strains, being predominantly detected in AICU and mostly related to MDR/XDR isolates, except for one isolate presenting MultiS phenotype. Two of nine isolates comprised clonal group H/ST244, where *bla*$_{\text{CTX-M1}}$ was shared by both isolates and two comprised clonal group I/ST244; the other five isolates were genetically unrelated and three harbored *bla*$_{\text{CTX-M1}}$ gene (Fig 2). Endemic clone in Brazil, ST277 is frequently associated to MDR/XDR isolates and dissemination of *bla*$_{\text{SPM-1}}$ gene [20, 23, 92]. Interestingly, isolates comprising clonal group A/ST277 shared *bla*$_{\text{SPM-1}}$, but

harbored distinct *bla*$_{OXA}$ genes; one isolate of clonal group B/ST277 harbored both *bla*$_{SPM-1}$ and *bla*$_{OXA-10}$ genes, while the other harbored only *bla*$_{OXA-10}$ gene; isolates comprising clonal group C/ST277 shared *bla*$_{SPM-1}$, but only one isolate harbored *bla*$_{OXA-2}$ (Fig 2). To our knowledge this is first report in Brazil of *P. aeruginosa* ST277 harboring both *bla*$_{SPM-1}$ and *bla*$_{OXA}$ variants genes.

Most of our isolates were identified as non-HCR, which among them non-resistant phenotypes (MultiS and ModR) were significantly predominant (*P* <0.0001) (Table 5), demonstrating that susceptible isolates are associated to higher clonal diversity [31]. Clonal group E/ST1284 comprised three isolates, which curiously, the only isolate harboring *bla*$_{CTX-M1}$ was negative for *exoU* gene, suggesting a reduction in virulence due to acquisition of a resistance marker (Fig 2). ST508 isolate was the only non-HRC harboring a resistance gene (*bla*OXA$_{-2}$) (Fig 2). Novel ST2554 was identified as DLV of HRC ST274, belonging to CC274. High-risk CC/ST274 strains have been recently detected in Indonesian ICUs, but are still predominant among CF patients, demonstrating high occurrence of mutation in genes related to antibiotic resistance [93, 94, 95]. ST2554 isolate presented a ModR phenotype and *exoS*$^{+}$/*exoU*$^{+}$ virulotype, also indicating acquisition of PAPI-2 containing *exoU* gene during divergence from ST274 (Fig 2). In spite of the non-detection of ST274 among our isolates, it may not rule out the presence of this genotype in the hospital, as we observe a more direct genetic relationship between both strains.

Certain clonal strains are expected to be linked to T3SS virulotypes. Recent phylogenetic analysis by Sawa et al. 2020 [19] attributed *exoU*+ virulotype to ST253, possibly explaining the highest virulence potential of ST235 observed clinical studies [18, 23]. Also, Sanchez-Diener et al. (2017) [72] on animal model based study showed that lower

virulence was linked to XDR profiles, which are typically found among HRC. However, different virulence potentials were observed among HRC, being higher for ST111 and ST235 and lower for ST175. Our MLST lineages were not significantly associated to specific T3SS virulotypes (P =0.5820) (Table 5), however, we observed the presence of *exoS*$^{+}$/*exoU*$^{+}$ virulotype among the four detected HRC. Moreover, we observed isolates HRC ST244 and non-HRC ST508 harboring *exoS*$^{+}$/*exoU*$^{+}$ virulotype, while they were expected to be linked the invasive *exoS*$^{+}$ virulotype as recently reported [19]. Therefore, our findings highlight the importance of virulence genotyping and suggest the local acquisition of PAPI-2 containing *exoU* gene by HRC, which could worsen the local clinical scenario as most of HCR were also MDR/XDR.

The elevated virulence and resistance potential presented by our isolates might negatively affect patient's outcomes, however the present study did not perform associations to clinical and epidemiological data due to unavailability of patients records. Moreover, we did not perform phenotypic tests evaluating secretion/expression of virulence factors such as elastases, proteases, T3SS exotoxins, QS molecules and the sequencing of the virulence genes, thus preventing a better establishment of genotype-phenotype relationships. Finally, the non-characterization of hospital environment isolates may have underestimated the genetic background in the study site, also impairing analysis of transmission from in-hospital environmental reservoirs. Studies comprising a larger number of clinical and environmental isolates from the study site and from other hospitals are highly recommended in order to better establish *P. aeruginosa* transmission in the region, as well as, relationships between of virulence, antibiotic susceptibility and genetic diversity.

Conclusions

To conclude, this is the first study deeply exploring virulence, antibiotic resistance and genetic background of *P. aeruginosa* isolates from ICUs at specialized hospital in the Brazilian Amazon region. Herein, we reported a high prevalence of virulence-related markers, particularly of the rare $exoS^{+}/exoU^{+}$ virulotype, which may be associated to a high virulence and cytotoxic potential and transmission of genetic elements containing *exoU* gene among local isolates. It was also observed high percentages of resistance to different classes of antibiotics, particularly to carbapenems, in addition to MDR/XDR isolates harboring resistance genetic elements, alerting to a possible dissemination of these mechanisms and of isolates acting as resistance reservoirs. The importance genotyping by rep-PCR and MLST techniques in the surveillance of in-hospital isolates was noteworthy, which demonstrated circulation of MDR/XDR HRC clones, emergence of novel genetic lineages and a wide genetic diversity of strains circulating in different ICUs. Finally, our findings may be useful on comprehension of complex mechanisms of pathogenicity process, treatment guidance and establishment of strategies to control the spread of epidemic *P. aeruginosa* strains with a high lethality potential.

Acknowledgments

We thank Dra. Ana Roberta Fusco da Costa and Msc. Alex Brito Souza for the technical assistance in the MLST sequencing of samples.

The present study will be presented by Yan Corrêa Rodrigues to obtain his Ph.D. within the doctoral programme ‘Parasitic biology in Amazon Region’ at the State University of Pará, Brazil.

References

1. Hassuna NA, Mandour SA, Mohamed ES. Virulence Constitution of Multi-Drug-Resistant *Pseudomonas aeruginosa* in Upper Egypt. Infect Drug Resist. 2020;13:587-95.
2. Braga IA, Campos PA, Gontijo-Filho PP, Ribas RM. Multi-hospital point prevalence study of healthcare-associated infections in 28 adult intensive care units in Brazil. J Hosp Infect. 2018;99(3):318-24.
3. Berezin EN, Solorzano F, Latin America Working Group on Bacterial R. Gram-negative infections in pediatric and neonatal intensive care units of Latin America. J Infect Dev Ctries. 2014;8(8):942-53.
4. Luna CM, Rodriguez-Noriega E, Bavestrello L, Guzman-Blanco M. Gram-negative infections in adult intensive care units of latin america and the Caribbean. Crit Care Res Pract. 2014;2014:480463.
5. Haque M, Sartelli M, McKimm J, Abu Bakar M. Health care-associated infections - an overview. Infect Drug Resist. 2018;11:2321-33.
6. Russotto V, Cortegiani A, Graziano G, Saporito L, Raineri SM, Mammina C, et al. Bloodstream infections in intensive care unit patients: distribution and antibiotic resistance of bacteria. Infect Drug Resist. 2015;8:287-96.
7. Suarez C, Pena C, Tubau F, Gavalda L, Manzur A, Dominguez MA, et al. Clinical impact of imipenem-resistant *Pseudomonas aeruginosa* bloodstream infections. J Infect. 2009;58(4):285-90.
8. Rodrigues YC, Sarges ESNF, Conceição ML, Matos ECO, Gomes NJP, Quaresma AJG, Lima KVB. Infecções por *Pseudomonas aeruginosa*: aspectos clínicos,

microbiológicos e moleculares. In: Salgado YCS, organizer. Patologia das doenças. 4th ed. Ponta Grossa (PR): Atena Editora; 2018. pp. 53-69. Portuguese.

9. Cho HH, Kwon KC, Kim S, Koo SH. Correlation between virulence genotype and fluoroquinolone resistance in carbapenem-resistant *Pseudomonas aeruginosa*. Ann Lab Med. 2014;34(4):286-92.
10. Gellatly SL, Hancock RE. *Pseudomonas aeruginosa*: new insights into pathogenesis and host defenses. Pathog Dis. 2013;67(3):159-73.
11. Jabalameli F, Mirsalehian A, Khoramian B, Aligholi M, Khoramrooz SS, Asadollahi P, et al. Evaluation of biofilm production and characterization of genes encoding type III secretion system among *Pseudomonas aeruginosa* isolated from burn patients. Burns. 2012;38(8):1192-7.
12. Senturk S, Ulusoy S, Bosgelmez-Tinaz G, Yagci A. Quorum sensing and virulence of *Pseudomonas aeruginosa* during urinary tract infections. J Infect Dev Ctries. 2012;6(6):501-7.
13. Bradbury RS, Roddam LF, Merritt A, Reid DW, Champion AC. Virulence gene distribution in clinical, nosocomial and environmental isolates of *Pseudomonas aeruginosa*. J Med Microbiol. 2010;59(Pt 8):881-90.
14. Lombardi C, Tolchard J, Bouillot S, Signor L, Gebus C, Liebl D, et al. Structural and Functional Characterization of the Type Three Secretion System (T3SS) Needle of *Pseudomonas aeruginosa*. Front Microbiol. 2019;10:573.
15. Horna G, Amaro C, Palacios A, Guerra H, Ruiz J. High frequency of the exoU+/exoS+ genotype associated with multidrug-resistant "high-risk clones" of *Pseudomonas aeruginosa* clinical isolates from Peruvian hospitals. Sci Rep. 2019;9(1):10874.

16. Sawa T, Shimizu M, Moriyama K, Wiener-Kronish JP. Association between *Pseudomonas aeruginosa* type III secretion, antibiotic resistance, and clinical outcome: a review. Crit Care. 2014;18(6):668.
17. Hauser AR. The type III secretion system of *Pseudomonas aeruginosa*: infection by injection. Nat Rev Microbiol. 2009;7(9):654-65.
18. Pena C, Cabot G, Gomez-Zorrilla S, Zamorano L, Ocampo-Sosa A, Murillas J, et al. Influence of virulence genotype and resistance profile in the mortality of *Pseudomonas aeruginosa* bloodstream infections. Clin Infect Dis. 2015;60(4):539-48.
19. Sawa T, Momiyama K, Mihara T, Kainuma A, Kinoshita M, Moriyama K. Molecular epidemiology of clinically high-risk *Pseudomonas aeruginosa* strains: Practical overview. Microbiol Immunol. 2020;64(5):331-44.
20. de Oliveira Santos IC, Pereira de Andrade NF, da Conceicao Neto OC, da Costa BS, de Andrade Marques E, Rocha-de-Souza CM, et al. Epidemiology and antibiotic resistance trends in clinical isolates of *Pseudomonas aeruginosa* from Rio de janeiro - Brazil: Importance of mutational mechanisms over the years (1995-2015). Infect Genet Evol. 2019;73:411-5.
21. Matos ECO, Andriolo RB, Rodrigues YC, Lima PDL, Carneiro I, Lima KVB. Mortality in patients with multidrug-resistant *Pseudomonas aeruginosa* infections: a meta-analysis. Rev Soc Bras Med Trop. 2018;51(4):415-20.
22. Hong DJ, Bae IK, Jang IH, Jeong SH, Kang HK, Lee K. Epidemiology and Characteristics of Metallo-beta-Lactamase-Producing *Pseudomonas aeruginosa*. Infect Chemother. 2015;47(2):81-97.

23. Oliver A, Mulet X, Lopez-Causape C, Juan C. The increasing threat of *Pseudomonas aeruginosa* high-risk clones. Drug Resist Updat. 2015;21-22:41-59.
24. Yezli S, Shibl AM, Memish ZA. The molecular basis of beta-lactamase production in Gram-negative bacteria from Saudi Arabia. J Med Microbiol. 2015;64(Pt 2):127-36.
25. Martins AF, Zavascki AP, Gaspareto PB, Barth AL. Dissemination of *Pseudomonas aeruginosa* producing SPM-1-like and IMP-1-like metallo-beta-lactamases in hospitals from southern Brazil. Infection. 2007;35(6):457-60.
26. Livermore DM. Multiple mechanisms of antimicrobial resistance in *Pseudomonas aeruginosa*: our worst nightmare? Clin Infect Dis. 2002;34(5):634-40.
27. Liu H, Kong W, Yang W, Chen G, Liang H, Zhang Y. Multilocus sequence typing and variations in the oprD gene of *Pseudomonas aeruginosa* isolated from a hospital in China. Infect Drug Resist. 2018;11:45-54
28. Maâtallah M, Bakhrouf A, Habeeb MA, Turlej-Rogacka A, Iversen A, Pourcel C, et al. Four genotyping schemes for phylogenetic analysis of *Pseudomonas aeruginosa*: comparison of their congruence with multi-locus sequence typing. PLoS One. 2013 ;8(12):e82069. Erratum in: PLoS One. 2015;10(9):e0137931.
29. Achtman M. Evolution, population structure, and phylogeography of genetically monomorphic bacterial pathogens. Annu Rev Microbiol. 2008;62:53-70.
30. Feil EJ, Enright MC. Analyses of clonality and the evolution of bacterial pathogens. Curr Opin Microbiol. 2004;7(3):308-13.
31. Mulet X, Cabot G, Ocampo-Sosa AA, Dominguez MA, Zamorano L, Juan C, et al. Biological markers of *Pseudomonas aeruginosa* epidemic high-risk clones. Antimicrob Agents Chemother. 2013;57(11):5527-35.

32. Woodford N, Turton JF, Livermore DM. Multiresistant Gram-negative bacteria: the role of high-risk clones in the dissemination of antibiotic resistance. FEMS Microbiol Rev. 2011;35(5):736-55.

33. Wozniak DJ, Ohman DE. Transcriptional analysis of the *Pseudomonas aeruginosa* genes algR, algB, and algD reveals a hierarchy of alginate gene expression which is modulated by algT. J Bacteriol. 1994;176(19):6007-14.

34. Schurr MJ, Martin DW, Mudd MH, Deretic V. Gene cluster controlling conversion to alginate-overproducing phenotype in *Pseudomonas aeruginosa*: functional analysis in a heterologous host and role in the instability of mucoidy. J Bacteriol. 1994;176(11):3375-82.

35. Finnan S, Morrissey JP, O'Gara F, Boyd EF. Genome diversity of *Pseudomonas aeruginosa* isolates from cystic fibrosis patients and the hospital environment. J Clin Microbiol. 2004;42(12):5783-92.

36. Wolska K, Szweda P. Genetic features of clinical *Pseudomonas aeruginosa* strains. Pol J Microbiol. 2009;58(3):255-60.

37. Zhu H, Conibear TC, Bandara R, Aliwarga Y, Stapleton F, Willcox MD. Type III secretion system-associated toxins, proteases, serotypes, and antibiotic resistance of *Pseudomonas aeruginosa* isolates associated with keratitis. Curr Eye Res. 2006;31(4):297-306.

38. Sabharwal N, Dhall S, Chhibber S, Harjai K. Molecular detection of virulence genes as markers in *Pseudomonas aeruginosa* isolated from urinary tract infections. Int J Mol Epidemiol Genet. 2014;5(3):125-34.

39. Clinical and Laboratory Standards Institute. Performance Standards for Antimicrobial Susceptibility Testing: Approved Twenty-: Document M100-S28. Wayne, PA, USA: CLSI, 2018.

40. Magiorakos AP, Srinivasan A, Carey RB, Carmeli Y, Falagas ME, Giske CG, et al. Multidrug-resistant, extensively drug-resistant and pandrug-resistant bacteria: an international expert proposal for interim standard definitions for acquired resistance. Clin Microbiol Infect. 2012;18(3):268-81.

41. Bonnet R, Sampaio JL, Labia R, De Champs C, Sirot D, Chanal C, et al. A novel CTX-M beta-lactamase (CTX-M-8) in cefotaxime-resistant Enterobacteriaceae isolated in Brazil. Antimicrob Agents Chemother. 2000;44(7):1936-42.

42. Bert F, Branger C, Lambert-Zechovsky N. Identification of PSE and OXA beta-lactamase genes in *Pseudomonas aeruginosa* using PCR-restriction fragment length polymorphism. J Antimicrob Chemother. 2002;50(1):11-8.

43. Gonçalves DCPS, Lima ABM, Leão LSNO, Carmo Filho JR, Pimenta FC, Vieira JOG. Detecção de metalo-beta-lactamase em *Pseudomonas aeruginosa* isoladas de pacientes hospitalizados em Goiânia, Estado de Goiás. Rev Soc Bras Med Trop. 2009; 42:411-414. Portuguese.

44. Poirel L, Naas T, Nicolas D, Collet L, Bellais S, Cavallo JD, et al. Characterization of VIM-2, a carbapenem-hydrolyzing metallo-beta-lactamase and its plasmid- and integron-borne gene from a *Pseudomonas aeruginosa* clinical isolate in France. Antimicrob Agents Chemother. 2000;44(4):891-7.

45. Gales AC, Menezes LC, Silbert S, Sader HS. Dissemination in distinct Brazilian regions of an epidemic carbapenem-resistant *Pseudomonas aeruginosa* producing SPM metallo-beta-lactamase. J Antimicrob Chemother. 2003;52(4):699-702.

46. Nordmann P, Poirel L, Carrer A, Toleman MA, Walsh TR. How to detect NDM-1 producers. J Clin Microbiol. 2011;49(2):718-21.
47. Lomaestro BM, Tobin EH, Shang W, Gootz T. The spread of Klebsiella pneumoniae carbapenemase-producing K. pneumoniae to upstate New York. Clin Infect Dis. 2006;43(3):e26-8.
48. Curran B, Jonas D, Grundmann H, Pitt T, Dowson CG. Development of a multilocus sequence typing scheme for the opportunistic pathogen *Pseudomonas aeruginosa*. J Clin Microbiol. 2004;42(12):5644-9.
49. Francisco AP, Vaz C, Monteiro PT, Melo-Cristino J, Ramirez M, Carrico JA. PHYLOViZ: phylogenetic inference and data visualization for sequence based typing methods. BMC Bioinformatics. 2012;13:87.
50. Ayres M, Ayres Júnior M, Ayres DL, Santos AS. BioEstat 5.0. Aplicações estatísticas nas áreas das Ciências Bio-Médicas. Belém (PA): Instituto de Desenvolvimento Sustentável Mamirauá; 2008. Portuguese.
51. Brasil, Conselho Nacional de Saúde. Resolução Nº 466, de 12 de dezembro de 2012. Brasília. Avaliable from: http://conselho.saude.gov.br/resolucoes/2012/Reso466.pdf. Acesso em 14.May.2020.
52. Matos EC, Matos HJ, Conceicao ML, Rodrigues YC, Carneiro IC, Lima KV. Clinical and microbiological features of infections caused by *Pseudomonas aeruginosa* in patients hospitalized in intensive care units. Rev Soc Bras Med Trop. 2016;49(3):305-11.
53. Royer S, Faria AL, Seki LM, Chagas TP, Campos PA, Batistao DW, et al. Spread of multidrug-resistant *Acinetobacter baumannii* and *Pseudomonas aeruginosa* clones in

patients with ventilator-associated pneumonia in an adult intensive care unit at a university hospital. Braz J Infect Dis. 2015;19(4):350-7.

54. Kipnis E, Sawa T, Wiener-Kronish J. Targeting mechanisms of *Pseudomonas aeruginosa* pathogenesis. Med Mal Infect. 2006;36(2):78-91.
55. Morales-Espinosa R, Delgado G, Espinosa LF, Isselo D, Méndez JL, Rodriguez C, Miranda G, Cravioto A. Fingerprint Analysis and Identification of Strains ST309 as a Potential High Risk Clone in a *Pseudomonas aeruginosa* Population Isolated from Children with Bacteremia in Mexico City. Front Microbiol. 2017;8:313.
56. Ruiz-Roldan L, Belles A, Bueno J, Azcona-Gutierrez JM, Rojo-Bezares B, Torres C, et al. *Pseudomonas aeruginosa* Isolates from Spanish Children: Occurrence in Faecal Samples, Antimicrobial Resistance, Virulence, and Molecular Typing. Biomed Res Int. 2018;2018:8060178.
57. Fazeli N, Momtaz H. Virulence Gene Profiles of Multidrug-Resistant *Pseudomonas aeruginosa* Isolated From Iranian Hospital Infections. Iran Red Crescent Med J. 2014;16(10):e15722.
58. Stacey SD, Pritchett CL. *Pseudomonas aeruginosa* AlgU Contributes to Posttranscriptional Activity by Increasing rsmA Expression in a mucA22 Strain. J Bacteriol. 2016;198(13):1812-26.
59. Lomholt JA, Poulsen K, Kilian M. Epidemic population structure of *Pseudomonas aeruginosa*: evidence for a clone that is pathogenic to the eye and that has a distinct combination of virulence factors. Infect Immun. 2001;69(10):6284-95.
60. Ballok AE, O'Toole GA. Pouring salt on a wound: *Pseudomonas aeruginosa* virulence factors alter Na+ and Cl- flux in the lung. J Bacteriol. 2013;195(18):4013-9.

61. Laarman AJ, Bardoel BW, Ruyken M, Fernie J, Milder FJ, van Strijp JA, et al. *Pseudomonas aeruginosa* alkaline protease blocks complement activation via the classical and lectin pathways. J Immunol. 2012;188(1):386-93.
62. Mitov I, Strateva T, Markova B. Prevalence of virulence genes among bulgarian nosocomial and cystic fibrosis isolates of *Pseudomonas aeruginosa*. Braz J Microbiol. 2010;41(3):588-95.
63. Najafi K, Kafil HS, Shokrian S, Azimi S, Asgharzadeh M, Yousefi M, et al. Virulence genes and antibiotic resistance profile of *Pseudomonas aeruginosa* isolates in Northwest of Iran. Journal of Pure and Applied Microbiology. 2015:(9):383- 389.
64. Matsumoto K. Role of bacterial proteases in pseudomonal and serratial keratitis. Biol Chem. 2004;385(11):1007-16.
65. Du X, Youle RJ, FitzGerald DJ, Pastan I. Pseudomonas exotoxin A-mediated apoptosis is Bak dependent and preceded by the degradation of Mcl-1. Mol Cell Biol. 2010;30(14):3444-52.
66. Finlayson EA, Brown PD. Comparison of antibiotic resistance and virulence factors in pigmented and non-pigmented *Pseudomonas aeruginosa*. West Indian Med J. 2011;60(1):24-32.
67. Lau GW, Hassett DJ, Ran H, Kong F. The role of pyocyanin in *Pseudomonas aeruginosa* infection. Trends Mol Med. 2004;10(12):599-606.
68. Dekimpe V, Deziel E. Revisiting the quorum-sensing hierarchy in *Pseudomonas aeruginosa*: the transcriptional regulator RhlR regulates LasR-specific factors. Microbiology. 2009;155(Pt 3):712-23.

69. Aboushleib HM, Omar HM, Abozahra R, Elsheredy A, Baraka K. Correlation of quorum sensing and virulence factors in *Pseudomonas aeruginosa* isolates in Egypt. J Infect Dev Ctries. 2015;9(10):1091-9.
70. Hu P, Chen J, Chen Y, Zhou T, Xu X, Pei X. Molecular epidemiology, resistance, and virulence properties of *Pseudomonas aeruginosa* cross-colonization clonal isolates in the non-outbreak setting. Infect Genet Evol. 2017;55:288-96.
71. Gomez-Zorrilla S, Juan C, Cabot G, Camoez M, Tubau F, Oliver A, et al. Impact of multidrug resistance on the pathogenicity of *Pseudomonas aeruginosa*: in vitro and in vivo studies. Int J Antimicrob Agents. 2016;47(5):368-74.
72. Sánchez-Diener I, Zamorano L, López-Causapé C, Cabot G, Mulet X, Peña C, et al. Interplay among Resistance Profiles, High-Risk Clones, and Virulence in the Caenorhabditis elegans *Pseudomonas aeruginosa* Infection Model. Antimicrob Agents Chemother. 2017;61(12):e01586-17.
73. Pobiega M, Chmielarczyk A, Kozioł J, Pomorska-Wesolowska M, Ziolkowski G, Romaniszyn D, et al. Virulence factors genes and drug resistance in *Pseudomonas aeruginosa* strains derived from different forms of community and healthcare associated infections. Postępy Higieny i Medycyny Doświadczalnej. 2018;72:751-9.
74. Recio R, Sanchez-Diener I, Viedma E, Melendez-Carmona MA, Villa J, Orellana MA, et al. Pathogenic characteristics of *Pseudomonas aeruginosa* bacteraemia isolates in a high-endemicity setting for ST175 and ST235 high-risk clones. Eur J Clin Microbiol Infect Dis. 2020;39(4):671-8.
75. de Almeida Silva KCF, Calomino MA, Deutsch G, de Castilho SR, de Paula GR, Esper LMR, et al. Molecular characterization of multidrug-resistant (MDR) *Pseudomonas aeruginosa* isolated in a burn center. Burns. 2017;43(1):137-43.

76. Cacci LC, Chuster SG, Martins N, Carmo PR, Girão VB, Nouér SA, et al. Mechanisms of carbapenem resistance in endemic *Pseudomonas aeruginosa* isolates after an SPM-1 metallo-β-lactamase producing strain subsided in an intensive care unit of a teaching hospital in Brazil. Mem Inst Oswaldo Cruz. 2016;111(9):551-8..
77. Sader HS, Jones RN, Gales AC, Silva JB, Pignatari AC; SENTRY Participants Group (Latin America). SENTRY antimicrobial surveillance program report: Latin American and Brazilian results for 1997 through 2001. Braz J Infect Dis. 2004 Feb;8(1):25-79.
78. Pellegrino FL, Teixeira LM, Carvalho Md Mda G, Aranha Nouer S, Pinto De Oliveira M, Mello Sampaio JL, et al. Occurrence of a multidrug-resistant *Pseudomonas aeruginosa* clone in different hospitals in Rio de Janeiro, Brazil. J Clin Microbiol. 2002;40(7):2420-4.
79. Labarca JA, Salles MJ, Seas C, Guzman-Blanco M. Carbapenem resistance in *Pseudomonas aeruginosa* and *Acinetobacter baumannii* in the nosocomial setting in Latin America. Crit Rev Microbiol. 2016;42(2):276-92.
80. Hermes DM, Pormann Pitt C, Lutz L, Teixeira AB, Ribeiro VB, Netto B, et al. Evaluation of heteroresistance to polymyxin B among carbapenem-susceptible and -resistant *Pseudomonas aeruginosa*. J Med Microbiol. 2013;62(Pt 8):1184-1189.
81. Poirel L, Bonnin RA, Nordmann P. Genetic support and diversity of acquired extended-spectrum beta-lactamases in Gram-negative rods. Infect Genet Evol. 2012;12(5):883-93.
82. Bradford PA. Extended-spectrum beta-lactamases in the 21st century: characterization, epidemiology, and detection of this important resistance threat. Clin Microbiol Rev. 2001;14(4):933-51.

83. Cabot G, Lopez-Causape C, Ocampo-Sosa AA, Sommer LM, Dominguez MA, Zamorano L, et al. Deciphering the Resistome of the Widespread *Pseudomonas aeruginosa* Sequence Type 175 International High-Risk Clone through Whole-Genome Sequencing. Antimicrob Agents Chemother. 2016;60(12):7415-23.
84. Garey KW, Vo QP, Larocco MT, Gentry LO, Tam VH. Prevalence of type III secretion protein exoenzymes and antimicrobial susceptibility patterns from bloodstream isolates of patients with *Pseudomonas aeruginosa* bacteremia. J Chemother. 2008;20(6):714-20.
85. Cho HH, Kwon KC, Sung JY, Koo SH. Prevalence and genetic analysis of multidrug-resistant *Pseudomonas aeruginosa* ST235 isolated from a hospital in Korea, 2008-2012. Ann Clin Lab Sci. 2013;43(4):414-9.
86. Aguilar-Rodea P, Zuniga G, Rodriguez-Espino BA, Olivares Cervantes AL, Gamino Arroyo AE, Moreno-Espinosa S, et al. Identification of extensive drug resistant *Pseudomonas aeruginosa* strains: New clone ST1725 and high-risk clone ST233. PLoS One. 2017;12(3):e0172882.
87. Treepong P, Kos VN, Guyeux C, Blanc DS, Bertrand X, Valot B, et al. Global emergence of the widespread *Pseudomonas aeruginosa* ST235 clone. Clin Microbiol Infect. 2018;24(3):258-66.
88. Kos VN, Deraspe M, McLaughlin RE, Whiteaker JD, Roy PH, Alm RA, et al. The resistome of *Pseudomonas aeruginosa* in relationship to phenotypic susceptibility. Antimicrob Agents Chemother. 2015;59(1):427-36.
89. Del Barrio-Tofino E, Lopez-Causape C, Cabot G, Rivera A, Benito N, Segura C, et al. Erratum for del Barrio-Tofino et al., "Genomics and Susceptibility Profiles of

Extensively Drug-Resistant *Pseudomonas aeruginosa* Isolates from Spain". Antimicrob Agents Chemother. 2018;62(1).

90. Chen Y, Sun M, Wang M, Lu Y, Yan Z. Dissemination of IMP-6-producing *Pseudomonas aeruginosa* ST244 in multiple cities in China. Eur J Clin Microbiol Infect Dis. 2014;33(7):1181-7.
91. Miranda CC, de Filippis I, Pinto LH, Coelho-Souza T, Bianco K, Cacci LC, et al. Genotypic characteristics of multidrug-resistant *Pseudomonas aeruginosa* from hospital wastewater treatment plant in Rio de Janeiro, Brazil. J Appl Microbiol. 2015;118(6):1276-86.
92. Silveira MC, Rocha-de-Souza CM, Albano RM, de Oliveira Santos IC, Carvalho-Assef APD. Exploring the success of Brazilian endemic clone *Pseudomonas aeruginosa* ST277 and its association with the CRISPR-Cas system type I-C. BMC Genomics. 2020;21(1):255.
93. Pelegrin AC, Saharman YR, Griffon A, Palmieri M, Mirande C, Karuniawati A, et al. High-Risk International Clones of Carbapenem-Nonsusceptible *Pseudomonas aeruginosa* Endemic to Indonesian Intensive Care Units: Impact of a Multifaceted Infection Control Intervention Analyzed at the Genomic Level. mBio. 2019;10(6).
94. Lopez-Causape C, Sommer LM, Cabot G, Rubio R, Ocampo-Sosa AA, Johansen HK, et al. Evolution of the *Pseudomonas aeruginosa* mutational resistome in an international Cystic Fibrosis clone. Sci Rep. 2017;7(1):5555.
95. Parkins MD, Somayaji R, Waters VJ. Epidemiology, Biology, and Impact of Clonal *Pseudomonas aeruginosa* Infections in Cystic Fibrosis. Clin Microbiol Rev. 2018;31(4).

Supporting information

S1 Table. Origin, resistance, genotyping and virulence data of the 54 *P. aeruginosa* isolates from the referral hospital under study.

* Novel ST

[a] Novel *aroE* allele

ICU, intensive care unit; AICU, adult intensive care unit; PICU, pediatric intensive care unit; NICU, neonatal intensive care unit; RTI, respiratory tract infection; BSI, bloodstream infection; FEP, cefepime; IMP, imipenem; MER, meropenem; TZP, piperacillin+tazobactam; GN, gentamicin; CIP, ciprofloxacin; POL, polymyxin B; MultiS, susceptible to all tested antibiotics; ModR, moderately resistant; MDR, multi-drug resistant; XDR, extensively drug-resistant; ST, sequence type.

S1 Fig. Prevalence of virulence-associated genes in the 54 *P. aeruginosa* isolates from the referral hospital under study.

Figure 1_MST Click here to download Figure Fig1.TIFF.tif

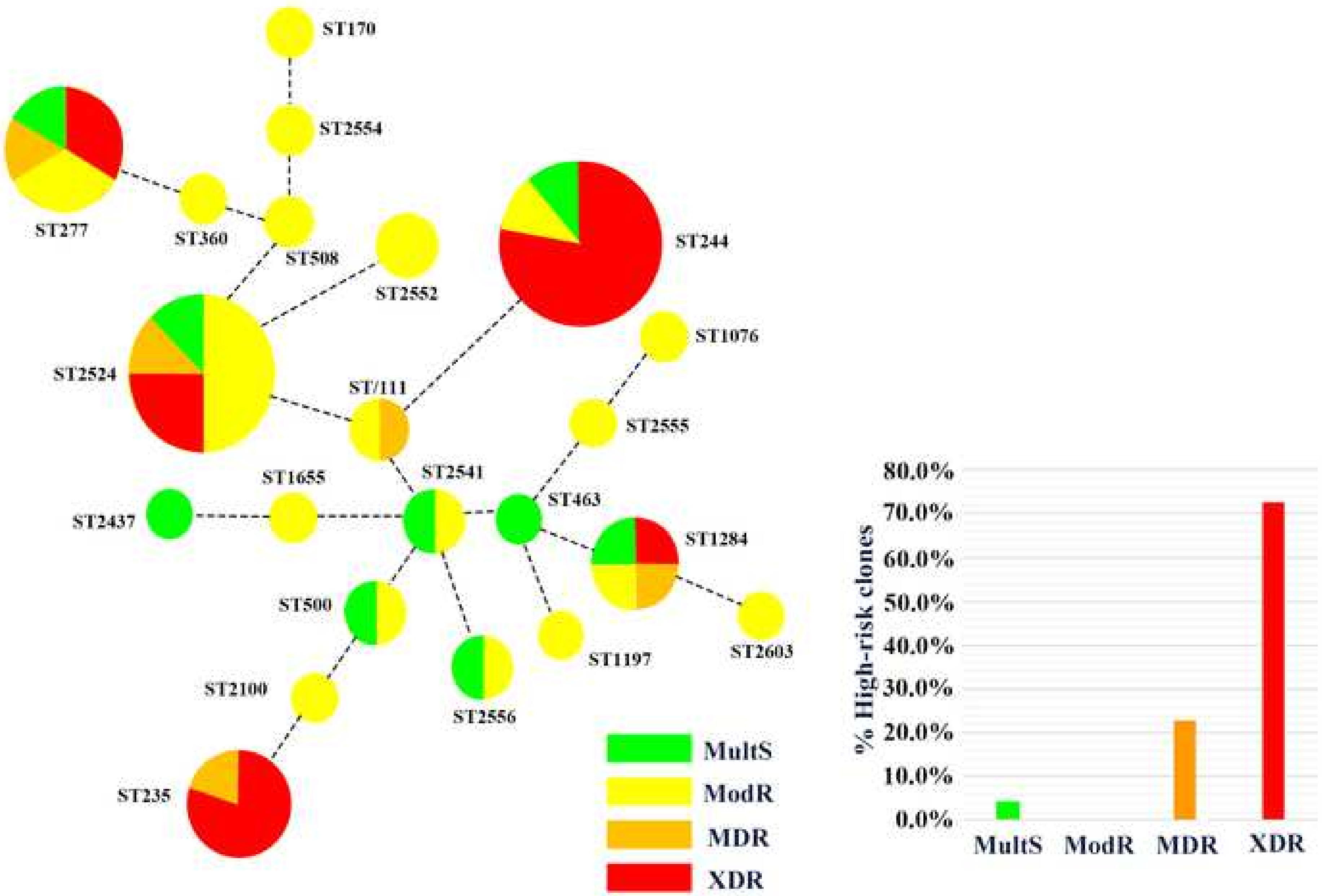

Figure 2_Dendrogram

Click here to download Figure Fig2.TIFF.tif

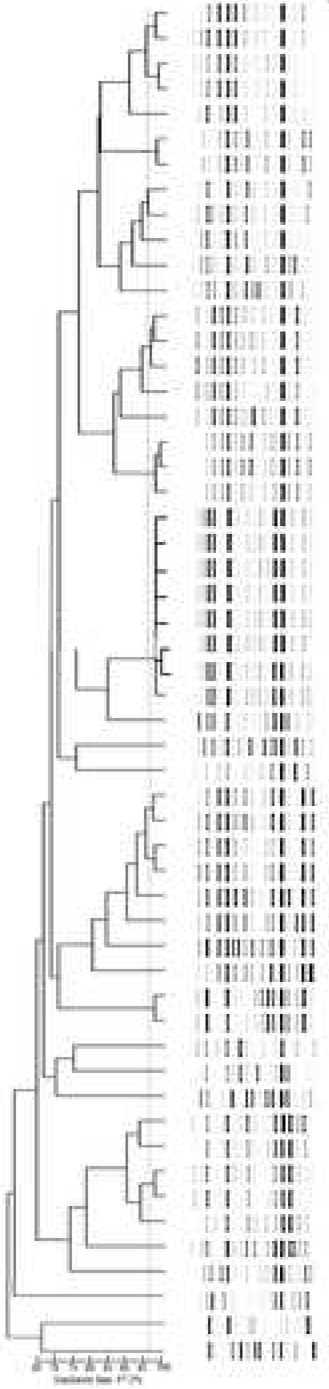

Id	Clonal group / Pattern	ST	Year	ICU	Clinical source	T3SS virulotype	Susceptibility phenotype	β-lactamase gene
SC060	A	277	2011	PICU	RTI	*exoS*$^-$/*exoU*$^+$	MDR	*bla* $_{SPM-1}$, *bla* $_{OXA-10}$
SC083	A	277	2011	AICU	RTI	*exoS*$^+$/*exoU*$^-$	XDR	*bla* $_{SPM-1}$, *bla* $_{OXA-2}$
SC007	B	277	2010	PICU	Other	*exoS*$^-$/*exoU*$^+$	XDR	*bla* $_{SPM-1}$, *bla* $_{OXA-10}$
SC016	B	277	2010	AICU	RTI	*exoS*$^-$/*exoU*$^+$	MDR	*bla* $_{OXA-10}$
SC015		1197	2010	AICU	Other	*exoS*$^-$/*exoU*$^+$	ModR	
SC115	C	277	2012	PICU	BSI	*exoS*$^+$/*exoU*$^-$	XDR	*bla* $_{SPM-1}$, *bla* $_{OXA-2}$
SC139	C	277	2012	PICU	RTI	*exoS*$^+$/*exoU*$^-$	XDR	*bla* $_{SPM-1}$
SC047		500	2011	NICU	BSI	*exoS*$^+$/*exoU*$^-$	MultS	
SC059		500	2011	NICU	BSI	*exoS*$^+$/*exoU*$^-$	ModR	
SC061		170	2011	AICU	RTI	*exoS*$^+$/*exoU*$^-$	ModR	
SC103		2554*	2012	AICU	BSI	*exoS*$^-$/*exoU*$^+$	ModR	
SC101		1655	2011	AICU	RTI	*exoS*$^-$/*exoU*$^+$	ModR	
SC037	D	235	2010	AICU	Other	*exoS*$^+$/*exoU*$^-$	XDR	*bla* $_{CTX-M2}$
SC033	D	235	2010	AICU	RTI	*exoS*$^-$/*exoU*$^+$	XDR	*bla* $_{CTX-M2}$
SC099	D	235	2011	PICU	Other	*exoS*$^-$/*exoU*$^+$	XDR	*bla* $_{CTX-M2}$
SC008		235	2010	AICU	Other	*exoS*$^-$/*exoU*$^+$	MDR	*bla* $_{CTX-M2}$
SC129		235	2012	AICU	RTI	*exoS*$^-$/*exoU*$^+$	XDR	
SC145	E	1284	2012	PICU	Other	*exoS*$^-$/*exoU*$^+$	ModR	
SC187	E	1284	2013	PICU	RTI	*exoS*$^-$/*exoU*$^+$	ModR	
SC162	E	1284	2013	PICU	Other	*exoS*$^+$/*exoU*$^-$	ModR	*bla* $_{CTX-M2}$
SC080	F	2524*	2011	NICU	BSI	*exoS*$^+$/*exoU*$^-$	ModR	
SC082	F	2524*	2011	NICU	BSI	*exoS*$^+$/*exoU*$^-$	ModR	
SC079	F	2524*	2011	NICU	BSI	*exoS*$^+$/*exoU*$^-$	MultS	
SC081	F	2524*	2011	NICU	BSI	*exoS*$^+$/*exoU*$^-$	MultS	
SC084	F	2524*	2011	NICU	Other	*exoS*$^-$/*exoU*$^+$	ModR	
SC086	F	2524*	2011	AICU	Other	*exoS*$^-$/*exoU*$^+$	MDR	
SC090	F	2524*	2011	PICU	RTI	*exoS*$^-$/*exoU*$^+$	ModR	
SC097	F	2524*	2011	AICU	RTI	*exoS*$^-$/*exoU*$^+$	ModR	
SC116		1076	2012	PICU	RTI	*exoS*$^-$/*exoU*$^+$	ModR	
SC128		463	2012	PICU	BSI	*exoS*$^-$/*exoU*$^+$	MultS	
SC169		244	2013	NICU	BSI	*exoS*$^+$/*exoU*$^-$	MultS	
SC032	G	244	2010	AICU	Other	*exoS*$^-$/*exoU*$^+$	XDR	
SC065	G	244	2011	AICU	Other	*exoS*$^+$/*exoU*$^-$	XDR	
SC026	H	244	2010	AICU	Other	*exoS*$^-$/*exoU*$^+$	MDR	*bla* $_{CTX-M2}$
SC029	H	244	2010	AICU	BSI	*exoS*$^+$/*exoU*$^-$	XDR	*bla* $_{CTX-M2}$
SC058		244	2011	AICU	RTI	*exoS*$^+$/*exoU*$^-$	XDR	*bla* $_{CTX-M2}$
SC048		244	2011	AICU	RTI	*exoS*$^+$/*exoU*$^-$	XDR	
SC069		244	2011	AICU	RTI	*exoS*$^+$/*exoU*$^-$	XDR	*bla* $_{CTX-M2}$
SC094		244	2011	AICU	Other	*exoS*$^-$/*exoU*$^+$	XDR	*bla* $_{CTX-M2}$
SC092	I	111	2011	AICU	Other	*exoS*$^-$/*exoU*$^+$	XDR	
SC158	I	111	2013	AICU	RTI	*exoS*$^+$/*exoU*$^-$	MDR	*bla* $_{CTX-M2}$
SC156		2100	2013	PICU	Other	*exoS*$^-$/*exoU*$^+$	ModR	
SC157		2603*	2013	PICU	Other	*exoS*$^-$/*exoU*$^+$	ModR	
SC150		508	2013	NICU	BSI	*exoS*$^-$/*exoU*$^+$	ModR	*bla* $_{OXA-2}$
SC136		2556*	2012	NICU	BSI	*exoS*$^+$/*exoU*$^-$	MultS	
SC159		2556*	2013	NICU	BSI	*exoS*$^+$/*exoU*$^-$	ModR	
SC010	J	2552*	2010	AICU	RTI	*exoS*$^-$/*exoU*$^+$	ModR	
SC012	J	2552*	2010	AICU	RTI	*exoS*$^-$/*exoU*$^+$	MultS	
SC119		2437	2012	AICU	RTI	*exoS*$^-$/*exoU*$^+$	MultS	
SC163		1284	2013	AICU	RTI	*exoS*$^-$/*exoU*$^+$	ModR	
SC009		2541*	2010	AICU	RTI	*exoS*$^+$/*exoU*$^-$	ModR	
SC004		2541*	2010	AICU	RTI	*exoS*$^+$/*exoU*$^-$	MultS	
SC095		360	2012	PICU	RTI	*exoS*$^+$/*exoU*$^-$	ModR	
SC126		2555*	2012	NICU	Other	*exoS*$^-$/*exoU*$^+$	ModR	

Supporting Table 1

Click here to access/download
Supporting Information
S1_Table.xlsx.xlsx

Supporting Figure 1

Click here to access/download
Supporting Information
S1_Fig.TIFF.tif
