# Supplementary material for: High prevalence of atypical virulotype and genetically diverse background among Pseudomonas aeruginosa isolates from a referral hospital in the Brazilian Amazon
Source: PLoS One. 2020 Sep 10;15(9):e0238741. doi: 10.1371/journal.pone.0238741 (PMC7482967; doi:10.1371/journal.pone.0238741)
Supplement: Attachment — Submitted filename: Response to Reviewers.docx [file pone.0238741.s004.docx]

**To Dr. Grzegorz Woźniakowski,**

Academic Editor

PLOS ONE

Aug 03, 2020

Dear Dr. Grzegorz Woźniakowski,

After a careful review, we are pleased resubmitted the research article entitled “**High prevalence of atypical virulotype and genetically diverse background among *Pseudomonas aeruginosa* isolates from a referral hospital in the Brazilian Amazon**” for consideration for publication in Plos One. Academic Editor and reviewers’ suggestions were accepted and incorporated to a better presentation of the manuscript.

Thank you for considering!

Sincerely,

**Yan Corrêa Rodrigues – PhD candidate**

Programa de Pós-graduação em Biologia Parasitária na Amazônia, State University of Pará

Tv. Perebebuí, 2623. Belém, Pará, Brazil

yan.13@hotmail.com, +5591988835583

**Karla Valéria Batista Lima – PhD**

Evandro Chagas Institute

Br. 316, km 7, s/n. Ananindeua, Pará, Brazil

karlalima@iec.pa.gov.br, +559132142116

**Response to Reviewers**

**Journal Requirements:**

When submitting your revision, we need you to address these additional requirements:

1. Please ensure that your manuscript meets PLOS ONE's style requirements, including those for file naming.

***Answer: Style requirements and file naming were corrected as requested.***

**Review Comments to the Author:**

Reviewer #1: The manuscript is technically sound and the presented data support the conclusions. Experiments have been conducted properly with reasonable sample size. The conclusions were drawn appropriately based on the data presented. There are some items need editing corrections as shown as sticky notes in the attached PDF file.

***Answer: We appreciate the reviewer comments. Editing corrections were incorporated through the manuscript and highlighted in the ‘Revised Manuscript with Track Changes’.***

**General Comment:**

***Fig 2 was corrected and improved as requested by Reviewer #1. In addition, Fig 1 was corrected and improved as requested by others co-authors.***
